# CO_2_ Fixation to Prebiotic Intermediates over Heterogeneous Catalysts

**DOI:** 10.1021/acs.accounts.4c00151

**Published:** 2024-07-18

**Authors:** Youngdong Song, Harun Tüysüz

**Affiliations:** Department of Heterogeneous Catalysis, Max-Planck-Institut für Kohlenforschung, Kaiser-Wilhelm-Platz 1, 45470 Mülheim an der Ruhr, Germany

## Abstract

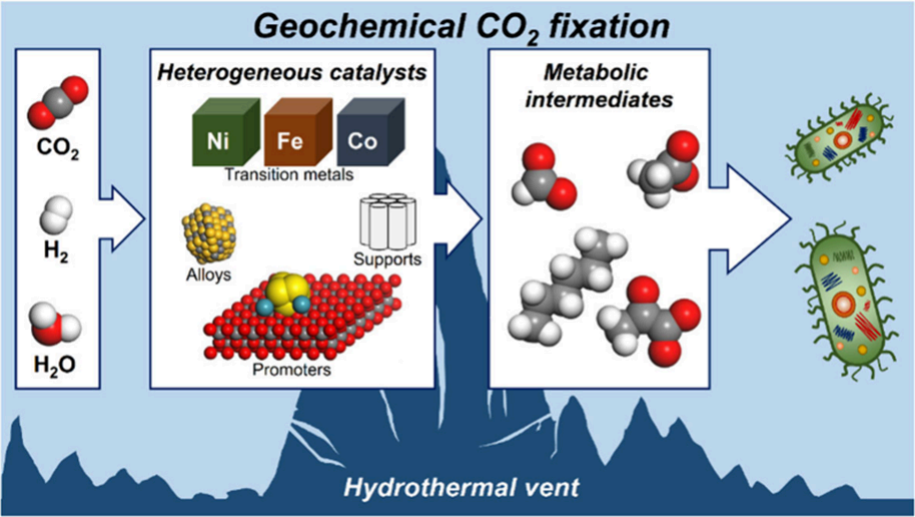

The study of the origin of life requires a multifaceted approach to understanding where and how life arose on Earth. One of the most compelling hypotheses is the chemosynthetic origin of life at hydrothermal vents, as this condition has been considered viable for early forms of life. The continuous production of H_2_ and heat by serpentinization generates reductive conditions at hydrothermal vents, in which CO_2_ can be used to build large biomolecules. Although this involves surface catalysis and an autocatalytic process, in which solid minerals act as catalysts in the conversion of CO_2_ to metabolically important organic molecules, the systematic investigation of heterogeneous catalysis to comprehend prebiotic chemistry at hydrothermal vents has not been undertaken.

In this Account, we discuss geochemical CO_2_ fixation to metabolic intermediates by synthetic minerals at hydrothermal vents from the perspective of heterogeneous catalysis. Ni and Fe are the most abundant transition metals at hydrothermal vents and occur in the active site of the enzymes carbon monoxide dehydrogenases/acetyl coenzyme A synthases (CODH/ACS). Synthetic free-standing NiFe alloy nanoparticles can convert CO_2_ to acetyl coenzyme A pathway intermediates such as formate, acetate, and pyruvate. The same alloy can further convert pyruvate to citramalate, which is essential in the biological citramalate pathway. Thermal treatment of Ni_3_Fe nanoparticles under NH_3_, which can occur in hydrothermal vents, results in Ni_3_FeN/Ni_3_Fe heterostructures. This catalyst has been demonstrated to produce prebiotic formamide and acetamide from CO_2_ and H_2_O using Ni_3_FeN/Ni_3_Fe as both substrate and catalyst. In the process of serpentinization, Co can be reduced in the vicinity of olivine, a Mg–Fe silicate mineral. This produces CoFe and CoFe_2_ with serpentine in nature, representing SiO_2_-supported CoFe alloys. In mimicking these natural minerals, synthetic SiO_2_-supported CoFe alloys demonstrate the same liquid products as NiFe alloys, namely, formate, acetate, and pyruvate under mild hydrothermal vent conditions. In contrast to the NiFe system, hydrocarbons up to C_6_ were detected in the gas phase, which is also present in hydrothermal vents. The addition of alkali and alkaline-earth metals to the catalysts results in enhanced formate concentration, playing a promotional role in CO_2_ reduction. Finally, Co was loaded onto ordered mesoporous SiO_2_ after modification with cations to simulate the minerals found in hydrothermal vents. These catalysts were then investigated under diminished H_2_O concentration, revealing the conversion of CO_2_ to CO, CH_4_, methanol, and acetate. Notably, the selectivity to metabolically relevant methanol was enhanced in the presence of cations that could generate and stabilize the methoxy intermediate. Calculation using the machine learning approach revealed the possibility of predicting the selectivity of CO_2_ fixation when modifying mesoporous SiO_2_ supports with heterocations. Our research demonstrates that minerals at hydrothermal vents can convert CO_2_ into metabolites under a variety of prebiotic conditions, potentially paving the way for modern biological CO_2_ fixation processes.

## Key References

- BeyazayT.; Ochoa-HernándezC.; SongY.; BelthleK. S.; MartinW. F.; TüysüzH.Influence of Composition of Nickel-Iron Nanoparticles for Abiotic CO_2_ Conversion to Early Prebiotic Organics. Angew. Chem., Int. Ed.2023, 62, e20221818910.1002/anie.20221818936951652.^1^*This article reports on the preparation of NiFe nanoparticles via the hard templating method and their role as synthetic solid catalysts for CO_2_ fixation to the acetyl-coenzyme A pathway intermediates such as formate, acetate, and pyruvate under hydrothermal vent conditions.*
- SongY.; BeyazayT.; TüysüzH.Effect of Alkali- and Alkaline-Earth-Metal Promoters on Silica-Supported Co–Fe Alloy for Autocatalytic CO_2_ Fixation. Angew. Chem., Int. Ed.2024, 63, e20231611010.1002/anie.20231611038127486.^2^*This article reveals the impact of the alkaline and alkaline-earth metal promoters on mesoporous SiO_2_-supported CoFe alloys for autocatalytic CO_2_ fixation to intermediates of the acetyl coenzyme A pathway and hydrocarbons up to C_6_.*
- BeyazayT.; BelthleK. S.; FarèsC.; PreinerM.; MoranJ.; MartinW. F.; TüysüzH.Ambient Temperature CO_2_ Fixation to Pyruvate and Subsequently to Citramalate over Iron and Nickel Nanoparticles. Nat. Commun.2023, 14, 57036732515 10.1038/s41467-023-36088-wPMC9894855.^3^*This study demonstrates stepwise CO_2_ fixation over FeNi nanoparticles. The Ni_3_Fe catalyst could convert CO_2_ to pyruvate and citramalate, which plays a key role in the biological citramalate pathway.*
- BeyazayT.; MartinW. F.; TüysüzH.Direct Synthesis of Formamide from CO_2_ and H_2_O with Nickel-Iron Nitride Heterostructures under Mild Hydrothermal Conditions. J. Am. Chem. Soc.2023, 145, 19768–1977937642297 10.1021/jacs.3c05412PMC7615090.^4^*This article reports on the preparation of NiFe nitride heterostructures, which act as substrates and catalysts that could convert CO_2_ and H_2_O to prebiotic formamide and acetamide.*

## 1 Introduction

Deciphering the origin of life on Earth is one of the most challenging tasks that humanity can undertake. We all instinctively want to know where and how we came to exist on Earth as we do today. More specifically, questions such as “What was the initial form of life?”, “Where did life first emerge?”, and “How did we evolve from the earliest form of life?” have been asked since ancient times. A wide range of research fields are involved in addressing these questions in a scientific manner including chemistry, biology, astronomy, and geology.^5^ Among these approaches, chemistry plays a crucial role by providing insights into molecular processes under primordial Earth conditions. In this regard, prebiotic chemistry tackles the synthesis of organic molecules and basic building blocks of life such as amino acids, fatty acids, and sugars from inorganic matter under probable primordial Earth conditions.^6^

Before discussing the origin of life in detail, we should define what life is. In a consensus, there are three basic requirements for life: (i) metabolism to obtain energy from surroundings to sustain itself, (ii) transmission of genetic information by self-replication, and (iii) compartmentalization by lipid cells to distinguish itself from the environment.^7,8^ Although we cannot pinpoint exactly when life arose on Earth, it is generally accepted that life first emerged 3.5–4.1 billion years ago based on fossil records, radiometric dating, and carbon isotope measurements.^9^ As the gap between the first life and current biological communities is immense, there have been several hypotheses/theories on the origin of life on Earth. Modern prebiotic chemistry is based on the pioneering studies from Oparin and Haldane in the 1920s and 1930s, where they suggested that life could have originated from simple organic molecules spontaneously synthesized under reducing conditions.^10^ This “prebiotic soup” theory was further supported by Miller’s experiment in 1953, where they were able to produce various amino acids from simple inorganic and organic molecules of H_2_, CH_4_, H_2_O, and NH_3_ by mimicking the reducing Earth conditions suggested by Oparin and Haldane.^11^ Although the current view postulates that prebiotic conditions were more oxidizing, they paved the way for the current prebiotic chemistry research.^12^ Currently, there are two main theories about the origin of life: the ribonucleic acid (RNA) world and metabolism first.^13^ The RNA world theory posits that RNA was the first biomolecule synthesized and it acts as an information transmitter and catalyst for simple chemical reactions. The metabolism-first theory, on the other hand, proposes that inorganic matter catalyzed a series of chemical reactions for the accumulation of organic molecules that preceded genetic transmission.^14,15^ These environments can be visualized in real life such as hot springs, meteorites, and hydrothermal vents.^16^

Deep-sea hydrothermal vents have gained a great deal of attention in the origin of life community since their first discovery in 1977.^17^ In the journey to discover hydrothermal vents, unanticipated biological communities have been found around hydrothermal vents, which means that the hydrothermal vent rich in chemicals can harbor microbial communities. This striking discovery has inspired the chemosynthetic origin of life at hydrothermal vents.^18,19^ They could provide information about the Earth’s primitive state, not only because they have existed since the Hadean (4.0–4.6 billion years ago), but also because they are likely environments for the emergence of life on Earth. With over 500 hydrothermal vents discovered to date, a wide range of physical and chemical conditions have been reported including pH, temperature, and chemicals.^20^ Hydrothermal vents are classified largely into two groups: black smokers and white smokers.^21^ Black smokers are characterized by high-temperature effluents (350–407 °C) because they are located directly above a magma chamber. The acidic effluents (pH 2–3) are rich in transition metals including Fe and Mn in sulfides. Along with gaseous inorganic and organic molecules (H_2_S, CO_2_, H_2_, and CH_4_), black smokers harbor biological communities. On the other hand, white smokers are located a few kilometers away from the magma chamber and provide a significantly different environment.^22^ Rich in Mg and Fe, olivine minerals in white smokers react with H_2_O, producing basic effluent (pH 9–11) and white precipitates.^23,24^ In addition, the warm outflow (40–120 °C) creates more viable conditions for life. Interestingly, the heat for warm outflow in white smokers is not supplied from the magma chamber as in black smokers. The exothermic reaction of ultramafic rock and H_2_O, called serpentinization, provides continuous heat and H_2_ in white smokers.^25^ This creates reductive conditions under which large organic molecules can be synthesized from inorganic CO_2_ or carbonate, implying that the first life on Earth could emerge at hydrothermal vents.

A variety of microbial communities currently inhabit hydrothermal vents. Notable microorganisms are acetogens and methanogens, which metabolize nourishing chemicals at hydrothermal vents including CO_2_, H_2_, formate, and acetate for anaerobic respiration and carbon fixation.^21,26^ They use the reductive acetyl coenzyme A (acetyl-CoA) pathway, also known as the Wood–Ljungdahl pathway, which is considered the most ancient because it takes place in the anaerobic conditions of primordial Earth and is found in both archaea and bacteria.^27^ In addition, the acetyl-CoA pathway is exergonic, producing adenosine triphosphate (ATP) that can facilitate other biochemical reactions.^28^ The linearity of the acetyl-CoA pathway also supports its antiquity, as it does not necessarily require pre-existing complex molecules. In the acetyl-CoA pathway, CO_2_ is reduced to CO and a methyl group is added to form acetyl-CoA. The key enzymes are carbon monoxide dehydrogenase (CODH) and acetyl-CoA synthase (ACS) with the methyl group donating cobalamin.^29^ Notably, the active sites of these metalloenzymes and cofactors contain transition metals such as Ni, Fe, and Co, which are abundant in hydrothermal vents.^18^ In addition, hydrogenase enzymes in acetogens exploit H_2_ to drive CO_2_ respiration, playing a similar role as H_2_ in the inorganic systems.^30^ Given the high probability that the acetyl-CoA pathway represents the most ancient metabolism, this enzyme and cofactor-driven biochemical process may have originated from geochemical CO_2_ fixation at hydrothermal vents.

The chemical reactions at hydrothermal vents can be seen as heterogeneously driven catalytic processes, in which solid minerals catalyze reactions of inorganic chemicals such as H_2_, CO_2_, and NH_3_ in both liquid and gaseous phases. Moreover, the organic products of these reactions could serve as reactants for subsequent reactions to build larger molecules, thus creating autocatalytic sets for the origin of life.^31,32^ Given the diversity of minerals present at hydrothermal vents, they may be involved in numerous reactions as heterogeneous catalysts. This underscores the need for a comprehensive understanding of prebiotic chemistry from the perspective of heterogeneous catalysts to properly study chemical reactions and catalysts at hydrothermal vents. This understanding involves evaluating catalysts and reactions not only for activity and selectivity but also for the reaction mechanisms leading to the resulting products. The structures of catalysts play a crucial role in determining their activity and selectivity. Factors such as metal alloys, support materials, and promoters should be considered. Porous chimneys at hydrothermal vents are also critical in heterogeneous catalysis. Porous supports offer a higher surface area, which accommodates more active sites and increases the reaction rate. Screening different reaction conditions, including temperature, pressure, time, and pH, facilitates encompassing different hydrothermal vent scenarios. This exploration aims to determine which conditions are more plausible for geochemical CO_2_ fixation.

In this Account, we present concepts and strategies for the development of synthetic solid catalysts and our research progress for CO_2_ fixation to metabolic intermediates under simulated hydrothermal vent conditions. First, the catalytic functionalities of two of the most abundant elements found at hydrothermal vents, Ni and Fe, are discussed. Second, the role of support–catalyst interactions and promoters for autocatalytic CO_2_ fixation is elaborated by simulating hydrothermal minerals, focusing on SiO_2_-supported CoFe alloys. Finally, Co catalysts supported on cation-modified mesoporous SiO_2_ for CO_2_ hydrogenation in the gas phase under diminished H_2_O concentration are discussed and presented.

## 2 Solid Catalyst Development Strategies for CO_2_ Fixation

The study of CO_2_ fixation has encompassed diverse heterogeneous catalysts, such as commercial metal powders, synthetic free-standing nanoparticles, and supported catalysts. Among these, commercial metal powders and ground meteorites represent the most direct forms of heterogeneous catalysts.^16,33^ However, catalysts prepared by these methods often exhibit irregular chemical composition and particle shape and size and an ill-defined surface. This inherent variability can pose challenges for systematic investigation, particularly considering that only surface sites are involved in heterogeneous catalysis.

Synthetic free-standing nanoparticles, however, offer advantages by providing a monodispersed size distribution and consistent catalytic activity through well-established synthetic methods. Various techniques, including precipitation, solvothermal, and templating methods, are used to synthesize free-standing nanoparticles. In the precipitation method, active metal salts are dissolved in a solution containing capping agents. The addition of a reducing agent (e.g., NaBH_4_) then triggers the formation of metallic nanoparticles. For example, Fe nanoparticles have been synthesized by dissolving FeSO_4_ in H_2_O and adding NaBH_4_ to the solution, and the resulting Fe nanoparticles could convert CO_2_ to formate and acetate under mild hydrothermal conditions.^34^ Solvothermal methods involve heating the solution of the metal precursor to an elevated temperature for crystallization. Ni_3_S_2_ nanoparticles synthesized by this means have been shown to exhibit activity toward CO_2_ conversion to formate.^35^ The hard templating method utilizes rigid porous materials as templates. Metal salts are then transformed into catalytically active metals within the pore confinement of templates through heat and chemical treatments. Selective removal of templates results in metal nanoparticles and a negative replica of the hard template. Porous carbon templated Ni_3_Fe nanoparticles have been shown to be an effective catalyst for CO_2_ fixation to formate, acetate, and pyruvate under simulated hydrothermal vent conditions.^19^

On the other hand, catalytically active metals can be incorporated into support materials to enhance their performance and stability. Oxide materials, such as SiO_2_ and Al_2_O_3_, are commonly employed as support materials owing to their high thermal and chemical stability. In supported catalysts, the performance depends on a variety of factors such as the particle size of active metals, porosity and textural parameters of support, acidity/basicity, and nature of metal–support interaction.^36,37^ SiO_2_-supported transition metals resemble the naturally occurring minerals during the serpentinization process at the hydrothermal vents.^38^ Many physicochemical properties of metal-supported solid catalysts can affect their performance for CO_2_ fixation to prebiotic intermediates. Some of these aspects are discussed below in sections 2.2 and 2.3.

CO_2_ fixation using heterogeneous catalysts is applicable to both liquid and gaseous phases. In the liquid phase, particularly in H_2_O, CO_2_ dissolves easily due to its high solubility (Henry’s constant, *K*_H_^0^ = 0.033 M·atm^–1^ at 298.15 K). The dissolved CO_2_ exists in equilibrium as three different species (H_2_CO_3_/HCO_3_^–^/CO_3_^2–^), and the distribution of these species is influenced by the pH of the reaction medium, which must be taken into account. In addition, certain CO_2_ fixation reactions are thermodynamically more favorable in the liquid phase.^39^ On the other hand, gas-phase CO_2_ fixation allows studies under diminished H_2_O concentration, which can be induced in pores found in hydrothermal vent minerals and dissolved salts. This condition can be simulated using a gas-phase continuous flow reactor and the introduction of promoters. Detailed investigations of these aspects are discussed and presented in the following sections.

### 2.1 NiFe-Based Nanoparticles for Geochemical CO_2_ Fixation

Ni and Fe appear in the active site of carbon monoxide dehydrogenases/acetyl-CoA synthases (CODH/ACS), facilitating the biological fixation of CO_2_ via the acetyl-CoA pathway.^29^ In hydrothermal vents, Ni and Fe stand out as two of the most abundant transition metals. It has been reported that both commercial Ni and Fe powders, as well as Ni_3_Fe alloy, can convert CO_2_ to metabolic intermediates under hydrothermal vent conditions.^19,33^ In our investigation, various compositions of free-standing NiFe alloy nanoparticles were investigated for CO_2_ fixation.^1^ These nanoparticles were meticulously prepared by the hard templating methods using tea leaves as a hard template. These catalysts, including monometallic Ni and Fe, were investigated under 25 bar of CO_2_ and H_2_ in a 3:2 ratio.

**Figure 1:**
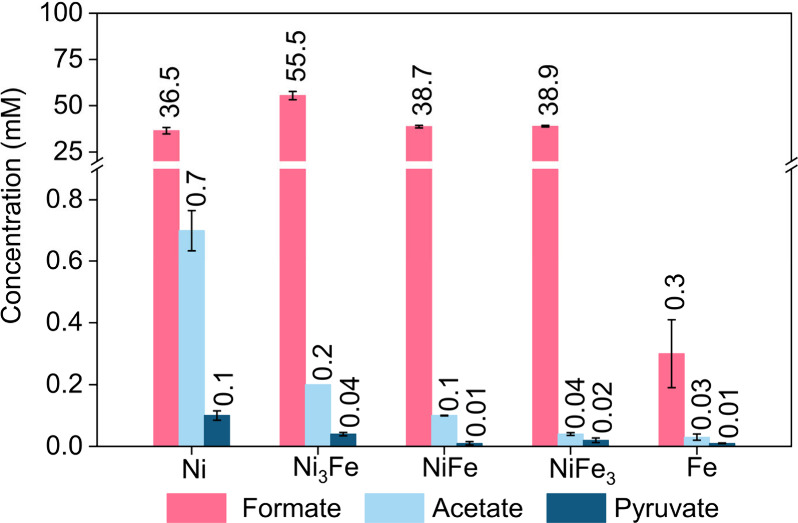
Product concentrations over Ni–Fe particles under 25 bar of CO_2_ + H_2_ gas mixture (CO_2_/H_2_ ratio is 3:2) at 100 °C for 24 h, based on HPLC analyses. The error bars were obtained from the standard deviations of three independent reactions. Adapted with permission from ref (1). Copyright 2023 Wiley-VCH Verlag GmbH & Co.

As shown in Figure 1, formate (C_1_), acetate (C_2_), and pyruvate (C_3_) were produced at 100 °C over NiFe alloy nanoparticles. These products are key for prebiotic CO_2_ fixation since they are the intermediates of the acetyl-CoA pathway. Among all the NiFe compositions tested, Ni_3_Fe exhibited the highest product concentration of 55.5 mM_formate_, 0.2 mM_acetate_, and 0.04 mM_pyruvate_. The concentration of products was much lower in the absence of H_2_ (1.1 mM_formate_, 0.03 mM_acetate_, and 0.02 mM_pyruvate_), implying that H_2_ is generated in situ by the reaction of Ni_3_Fe and H_2_O. This autocatalytic process is discussed in detail in section 2.2.

**Figure 2:**
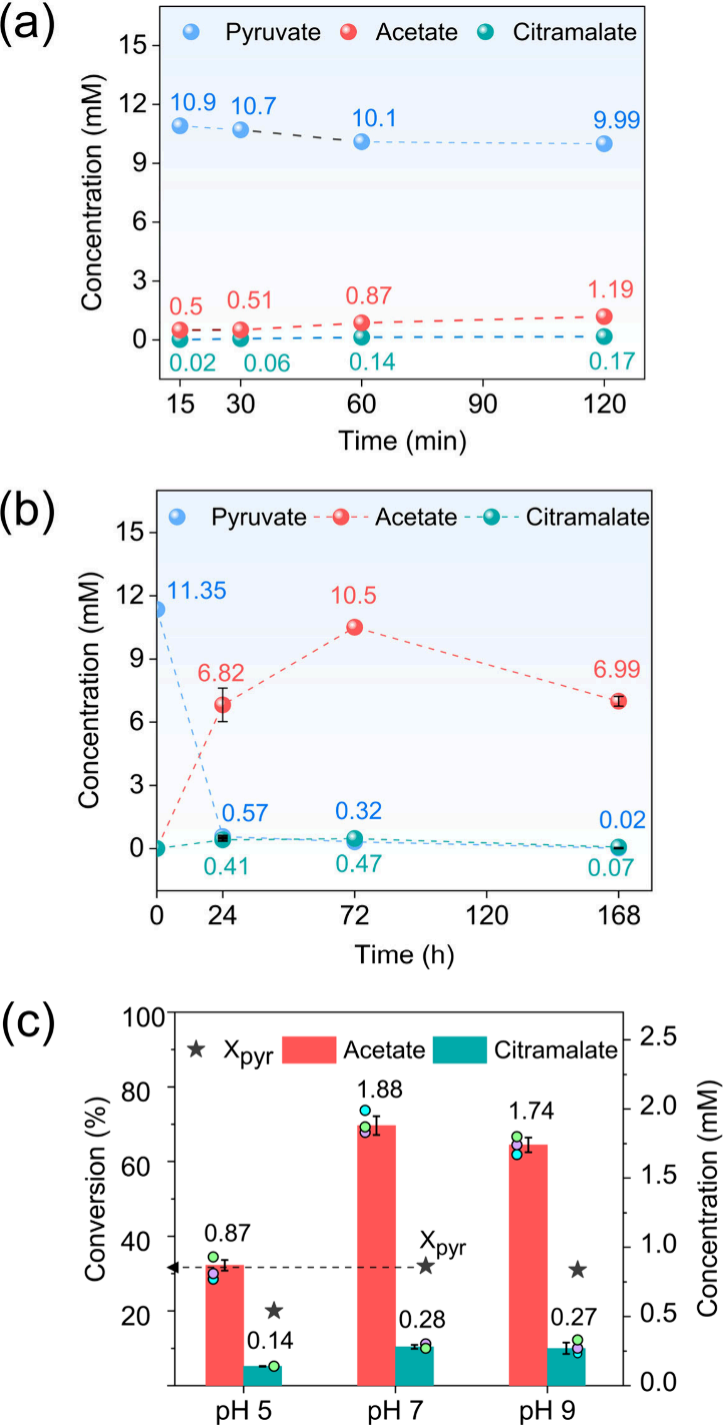
Product concentrations from pyruvate conversion (11.35 mM initial concentration) over Ni_3_Fe catalyst at 25 °C with different reaction times of (a) 15–120 min and (b) 24–168 h. (c) Pyruvate conversion and product concentrations with different starting pH values after 1 h at 25 °C over Ni_3_Fe. Pyruvate conversions are represented as *X*_pyr_ in the figure. Data in panels b and c are presented as mean values. Error bars correspond to the standard deviation of three independent reactions. Adapted with permission from ref (3). Copyright 2023 Springer Nature.

Motivated by the detection of metabolically crucial intermediates under simulated hydrothermal vent conditions, the most active Ni_3_Fe catalyst was further investigated for the conversion of pyruvate into metabolically relevant organic molecules.^3^ It was found that pyruvate could also be converted to acetate (0.87 mM), parapyruvate, and citramalate (0.14 mM) within 1 h. Citramalate was synthesized from pyruvate under physiological conditions in the presence of Ni_3_Fe catalyst. In biological systems, citramalate is typically produced from the reaction of pyruvate and acetyl-CoA catalyzed by citramalate synthase.^40^ An example of this is observed in *Rhodospirillum rubrum*, where acetate is assimilated through the citramalate pathway.^41^ Time-resolved reaction profiles for 2 h showed that 0.02 mM of citramalate was first detected after 15 min (Figure 2a). Extending the reaction time resulted in higher concentrations of acetate (10.5 mM) and citramalate (0.47 mM) over 72 h as shown in Figure 2b. Extension to 168 h resulted in lower concentrations of pyruvate and products, indicating their further decomposition to CO_2_. Variation of the pH revealed that the pyruvate conversion to citramalate is favorable under neutral and alkaline conditions (Figure 2c). Experiments with isotope-labeled reactants, such as ^12^C-acetate and ^13^C-pyruvate, revealed that citramalate is synthesized primarily by the formation of parapyruvate via the homoaldol condensation of pyruvate, followed by the subsequent decarboxylation of parapyruvate. The investigation of the catalyst after the reaction revealed that the surface of Ni_3_Fe was oxidized, while the bulk remained unchanged.

**Figure 3:**
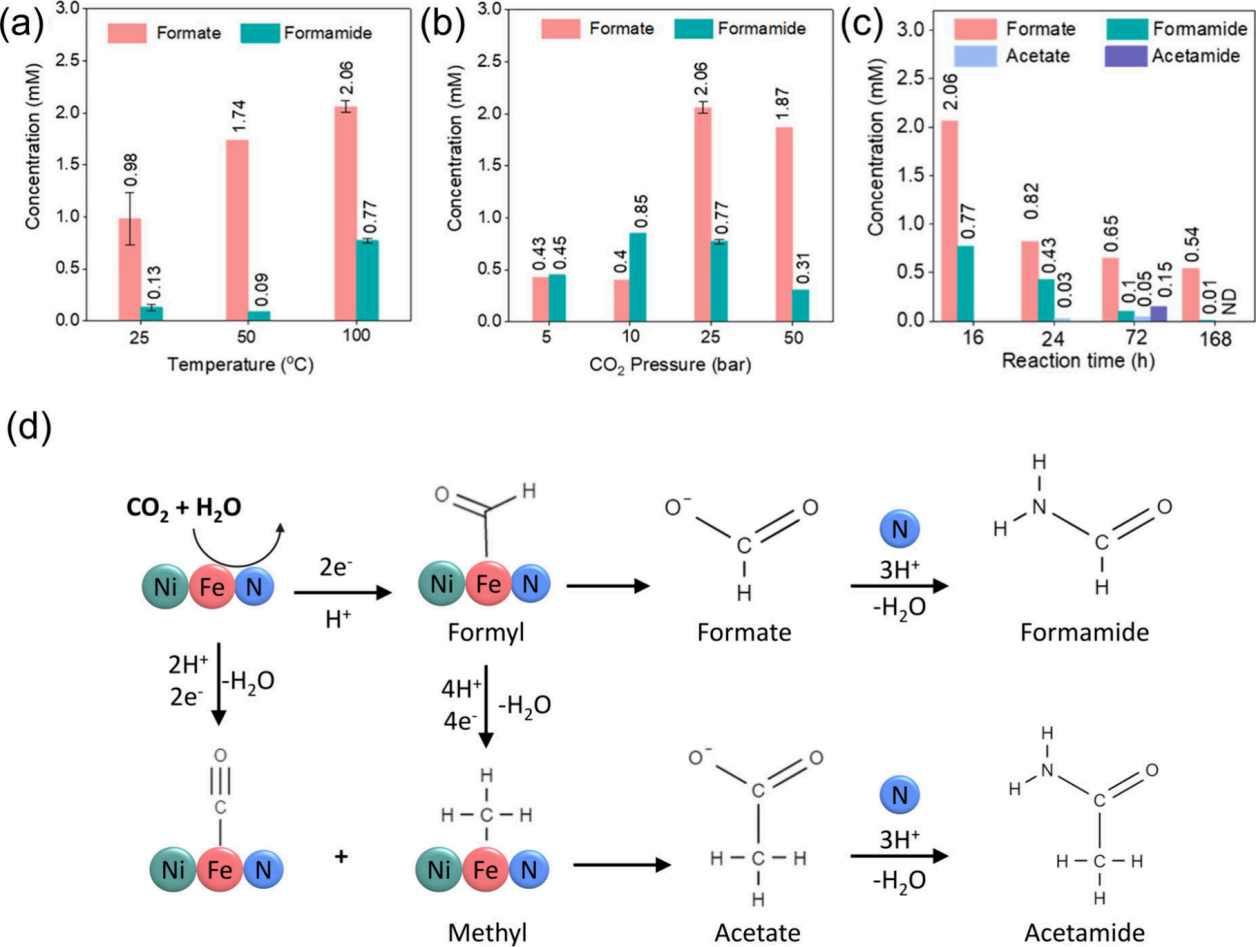
Concentrations of obtained products (a) at different temperatures under 25 bar, (b) under diverse initial CO_2_ pressures, and (c) after different reaction times. (d) Possible reaction pathway for the formation of amides from CO_2_ and H_2_O over the Ni_3_FeN/Ni_3_Fe. Error bars represent the standard deviations of at least two independent reactions. Adapted with permission from ref (4). Copyright 2023 American Chemical Society.

Although CO_2_ serves as a fundamental building block of life, nitrogen is also an essential element that forms amino acids. Nitrogen exists in the form of N_2_ and NH_3_ at hydrothermal vents, with NH_3_ being a reasonable nitrogen source due to the exorbitant stability of its triple bond (945 kJ·mol^–1^). When Ni_3_Fe was subjected to thermal treatment in the presence of ammonia, Ni_3_Fe underwent partial nitridation starting at 300 °C, resulting in a Ni_3_FeN/Ni_3_Fe heterostructure.^4^ We explored this heterostructure for catalytic CO_2_ fixation, in which we could detect the formation of formate and formamide in the presence of H_2_O. Formamide is considered a crucial precursor for prebiotic synthesis due to its ubiquity and ability to produce biologically relevant molecules such as adenine, purine, and uracil.^42^ Formamide production indicates the transfer of lattice nitrogen from Ni_3_FeN/Ni_3_Fe, demonstrating the dual role of Ni_3_FeN/Ni_3_Fe as both a catalyst and a substrate. In the temperature range of 25 to 100 °C (Figure 3a), formate and formamide concentrations were found to be proportional to temperature. Figure 3b displays a volcano plot of CO_2_ pressure with an optimum pressure of 25 bar, possibly due to the blockage of the active site under higher CO_2_ pressure. Extending the reaction time revealed the further conversion of formate and formamide to acetate and acetamide over 72 h, followed by the decomposition of these products due to high pressure and temperature in aqueous media, as shown in Figure 3c. Nitrogen was not detected in the catalyst structure after the reaction, indicating its consumption in the formation of formamide and acetamide. This phenomenon is consistent with the Mars–van Krevelen mechanism, where lattice-bound N in Ni_3_FeN/Ni_3_Fe leaves the surface as formamide and acetamide. In the proposed reaction pathway (Figure 3d), CO_2_ can be adsorbed on the surface of metals and reduced to *CO and the formyl group. The formyl group can be desorbed as formate or further reduced to acetate. Formate and acetate can react with lattice N in the heterostructure to form formamide and acetamide.

### 2.2 SiO_2_-Supported CoFe Minerals and the Effect of Promoters

During the serpentinization process, olivine minerals undergo a geochemical reaction with H_2_O, resulting in the formation of serpentine minerals and H_2_.^23^ Olivine minerals containing Mg–Fe silicates serve as the reactant in this reaction. The resulting H_2_ plays a significant role in the reduction of various transition metals, including Fe and Co, resulting in the formation of CoFe alloys supported on earth-abundant SiO_2_. The formation of wairauite (CoFe) and CoFe_2_ near serpentine supports the possible reduction of Fe and Co by serpentinization-induced H_2_.^43^ These SiO_2_-supported CoFe alloys can serve as heterogeneous catalysts for CO_2_ fixation under hydrothermal vent conditions.

**Figure 4:**
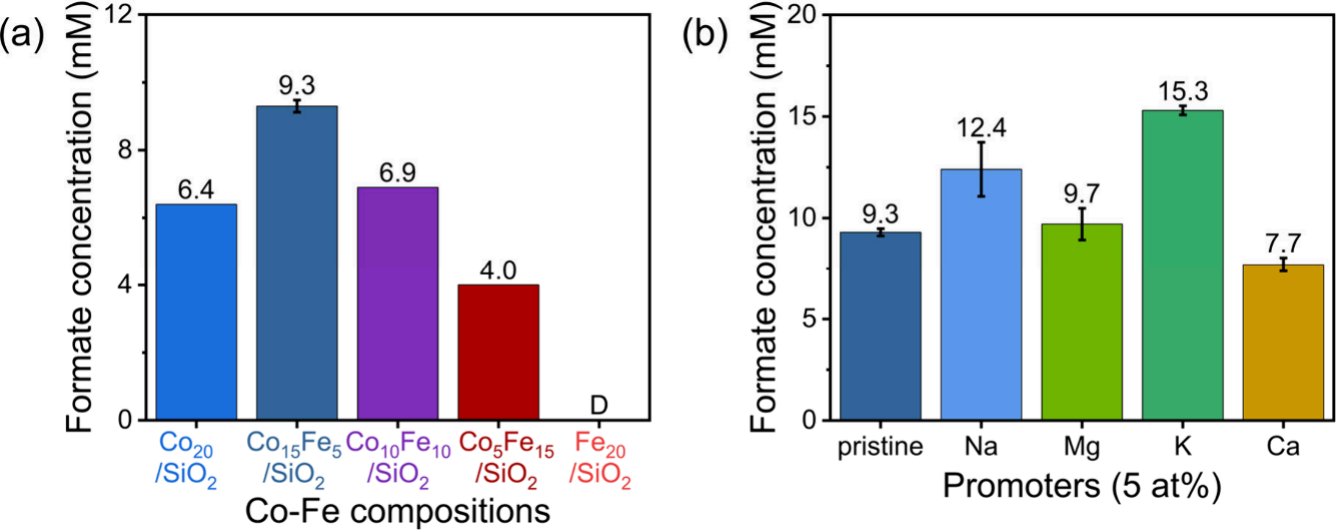
CO_2_ hydrogenation over SiO_2_-supported Co–Fe bimetallic catalysts. (a) Formate concentration with regard to Co–Fe composition on SiO_2_ with 20 atom % metal loading and (b) alkali- and alkaline-earth-metal promoters on Co_15_Fe_5_/SiO_2_. Error bars indicate standard deviations from triplicates. D corresponds to “detected”. *T* = 115 °C, *P* = 190 bar, *t* = 20 h. Adapted with permission from ref (2). Copyright 2024 Wiley-VCH Verlag GmbH & Co.

Previous studies have demonstrated the ability of a mixture of commercial Co and Fe powders to catalyze the conversion of NaHCO_3_, as a source of CO_2_, into long-chain hydrocarbons up to C_24_ at 300 °C.^43^ However, further investigations are needed to elucidate the potential of alloyed CoFe nanoparticles when incorporated into porous supports and alkali and alkaline earth metals under mild hydrothermal vent conditions. In the pursuit of understanding CO_2_ fixation over CoFe alloys within these conditions, we prepared SiO_2_-supported CoFe alloy structures using wet impregnation methods. The catalytic performances were evaluated in a closed autoclave system to simulate mild hydrothermal vent conditions at 115 °C and 190 bar of CO_2_. We have revealed that CO_2_ can be converted primarily to formate, acetate, and pyruvate. Importantly, the reduction of CO_2_ without external H_2_ suggests that H_2_ was produced in situ from H_2_O in the presence of catalysts. This phenomenon has been identified as an autocatalytic process, in which the products of a reaction act as reactants in subsequent reactions.^44^ The autocatalytic behavior of metals has been demonstrated with various metals including Fe, Mn, Zn, and Al.^45^ Generally, metals undergo oxidation to form metal oxides, accompanied by the production of H_2_ through reactions with H_2_O. The in situ generated H_2_ reduces the surface of the metal oxides, where the metal oxides themselves act as catalysts. For example, when the Fe metal reacts with H_2_O, it produces H_2_ and Fe_3_O_4_. The surface of Fe_3_O_4_ is partially reduced by in situ H_2_ to Fe_3_O_4–*x*_, which is the active surface for CO_2_ fixation. Similarly, CoFe alloys exhibit reactivity with H_2_O and CO_2_, resulting in the formation of (CoFe)CO_3_ and H_2_. The generation of in situ H_2_ facilitates the reduction of the (CoFe)CO_3_ surface, which could act as an autocatalyst to convert CO_2_ to metabolic intermediates. This autocatalytic process is essential in the context of prebiotic chemistry,^46^ as it contributes to the synthesis of metabolites in a prebiotic environment composed of CO_2_, H_2_, and H_2_O. In the gas phase, hydrocarbons up to C_6_ have been detected, as discussed in the Lost City hydrothermal field.^47^ The formation of C–C coupling products could be attributed to Fischer–Tropsch (FT) active CoFe alloys. Variation of CoFe compositions revealed that Co_15_Fe_5_ is the most active composition (Figure 4a). This trend resembles the free-standing NiFe alloy system discussed earlier, where Fe at a one-third ratio (i.e., Ni_3_Fe) showed the highest concentration of products.

**Figure 5:**
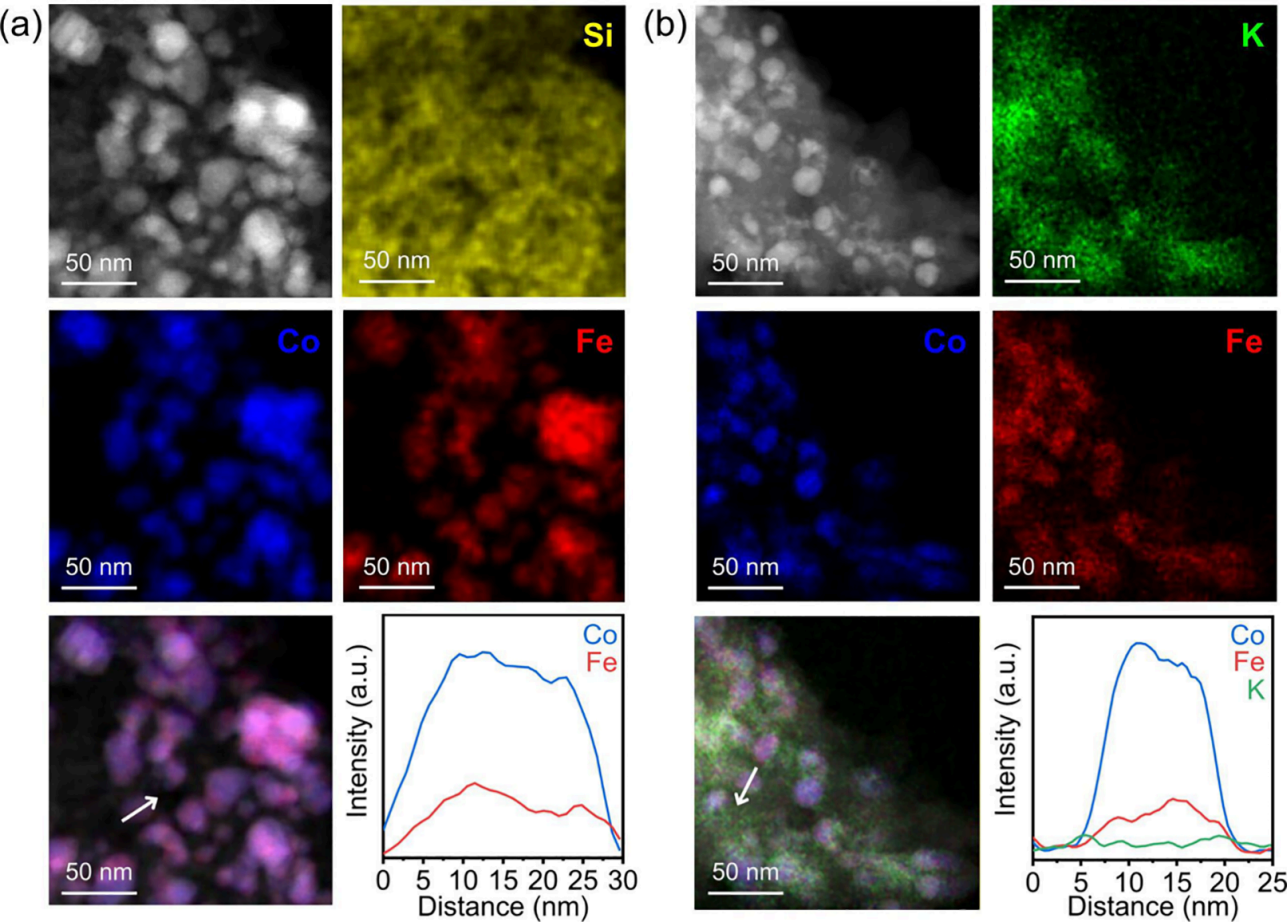
High-angle annular dark-field scanning transmission electron microscopy (HR-STEM) images and energy-dispersive X-ray spectroscopy (EDX) elemental mappings of two representative catalysts: (a) Co_15_Fe_5_/SiO_2_ and (b) Co_15_Fe_5_K_5_/SiO_2_. Line scan profiles are obtained from the white solid lines in the color-overlapped images. Reproduced with permission from ref (2). Copyright 2024 Wiley-VCH Verlag GmbH & Co.

Salts present in hydrothermal vents can play a significant role in CO_2_ fixation. Seawater contains significant concentrations of alkali and alkaline-earth metals such as K, Ca, Na, and Mg, which can promote CO_2_ hydrogenation. The introduction of K_2_O during the catalyst preparation step revealed significant effects on three key aspects: (1) the particle size of CoFe, (2) the pH of the reaction, and (3) the leaching of CoFe during the catalytic reaction. In Figure 5, the catalysts showed clear alloy formation of Co–Fe nanoparticles dispersed on the SiO_2_ support. Elemental mapping and line scanning through electron dispersive X-ray spectroscopy (EDX) proved that K was distributed over the entire surface of the catalysts. Notably, the average particle size of CoFe decreased from 36.5 to 14.0 nm with 5% K incorporation. This decrease in particle size contributed to CO_2_ fixation and the formation of a higher formate concentration, attributed to the increased surface area and the number of active sites. In addition, K elevated the pH of the reaction by partially dissolving in H_2_O. The pH was measured to increase from 5.8 to 10.5 with 5% K on the catalyst. Through systematic variations in K composition, it was found that a slightly basic pH favored the formate production due to the enhanced solubility of CO_2_. Analogous phenomena were observed in Cu and Mn systems, where bicarbonate ions (HCO_3_^–^), prevailing as the primary CO_2_ species in a slightly basic solution, functioned as the reactant for the production of formate.^45,48^

Expanding the scope to other alkali- and alkaline-earth-metal promoters, including Na, Mg, and Ca, we have shown a clear tendency where promotion by alkali metals outperformed that of alkaline-earth metals (Figure 4b). This could be attributed to the formation of insoluble hydroxides and carbonates, which consume CO_2_ that could otherwise be used for the reaction. The introduction of H_2_ into the reaction could provide insight into the reaction pathway, especially given its absence in the preceding stage. This results in a significant increase in the formate concentration from 9.3 to 72 mM, highlighting the dependence of CO_2_ reduction over the CoFe catalysts on H_2_ pressure. The reaction does not take place under a H_2_ atmosphere in the absence of the CoFe alloy, indicating that the CoFe alloy acts both as a reductant and as a catalyst.

### 2.3 Supported Co Nanoparticles for CO_2_ Fixation under Diminished H_2_O Concentration

The investigation of heterogeneous catalysis under hydrothermal vent conditions involves the presence of H_2_O in liquid-phase reactions. However, H_2_O presents a challenge in the synthesis of large molecules from CO_2_. Hydrolysis can break large molecules into smaller ones, posing a disadvantage for the polymerization of proteins or nucleic acids.^49^ Biological systems address this issue by enzymatically controlling hydrolysis. In hydrothermal vents, pores and salts can mimic such conditions. These conditions can be effectively simulated through gas-phase CO_2_ hydrogenation using a continuous flow reactor. In this system, H_2_O is restrictedly formed through the reverse water–gas shift reaction (CO_2_ + H_2_ ⇌ CO + H_2_O, Δ*H°* = 42.1 kJ mol^–1^).^50^ Subsequently, these molecules can be adsorbed on solid catalysts to produce various products such as alkane, alkene, methanol, and higher alcohols.

Co-based catalysts are widely studied for CO_2_ fixation owing to their activity toward Fischer–Tropsch processes. Given the vital role of Co in the Acetyl-CoA pathway, where it donates methyl groups,^2,51^ a deep understanding of CO_2_ fixation over Co catalysts implies significant importance. Metallic Co exhibits strong hydrogenation ability, predominantly yielding fully hydrogenated products, namely, CH_4_. On the other hand, partially reduced Co, such as Co_2_C and CoO, exhibit moderate hydrogenation activity, leading to the production of biologically relevant oxygenates.^52^ To assess the effect of cations present in hydrothermal vents on CO_2_ fixation under diminished H_2_O concentration, we designed synthetic minerals by loading Co nanoparticles into SBA-15 SiO_2_ and modified SBA-15 with common elements found at hydrothermal vents like Mg, Al, Ca, Ti, and Zr.^51^

**Figure 6:**
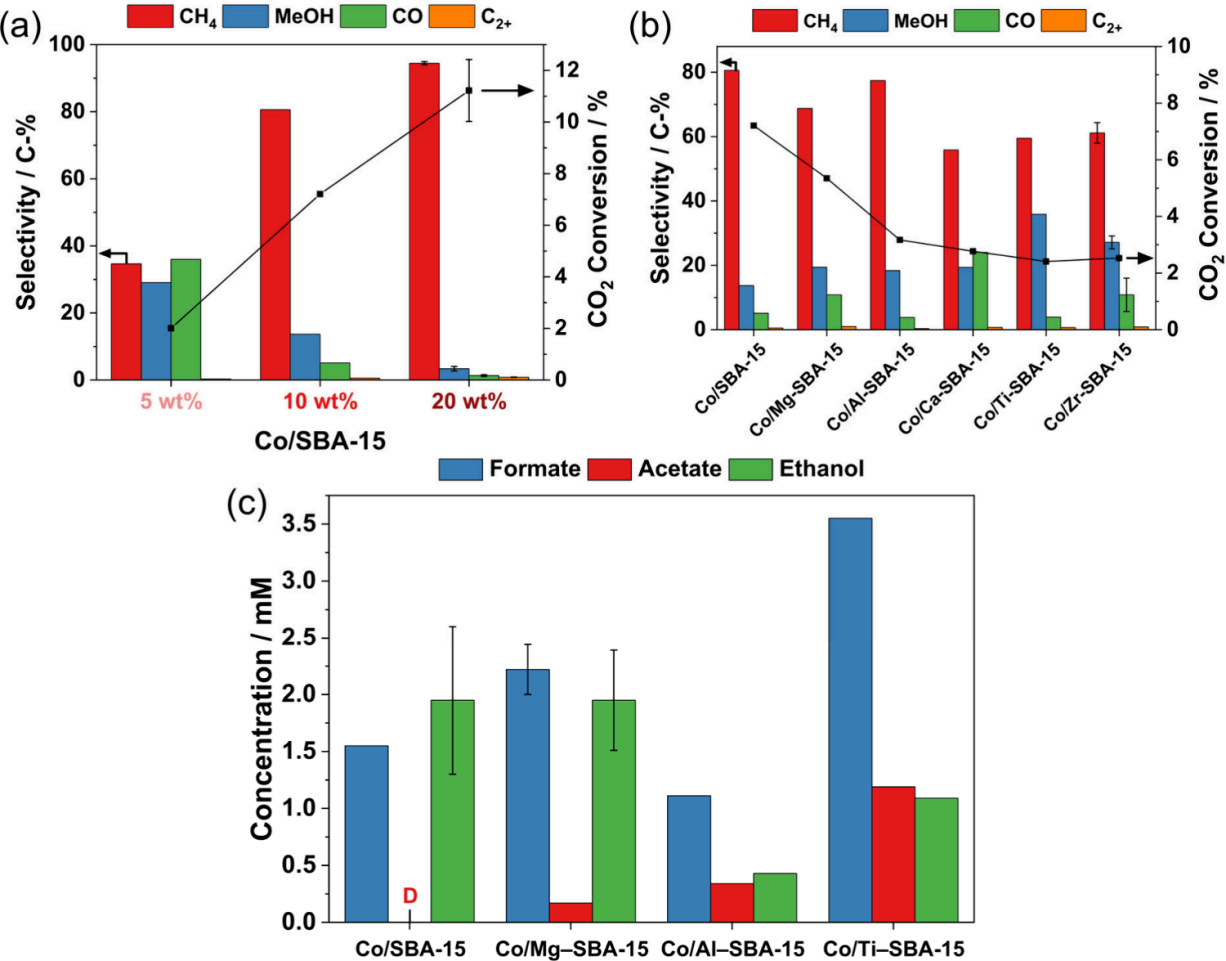
(a) Influence of Co-loading on the catalytic performance of Co/SBA-15 SiO_2_. (b) Catalytic conversion and product selectivity for CH_4_, methanol, CO, and C_2+_ hydrocarbons of 10 wt % Co/M–SBA-15 catalysts (M = Mg, Al, Ca, Ti, Zr). Conducted reaction conditions: *T* = 180 °C, *p* = 2.0 MPa, H_2_/CO_2_/Ar = 6:3:1, 4000 cm^3^ h^–1^ g_cat_^–1^, 36 h time-on-stream. Exemplary error bars are shown based on the reproduction of the reaction with different catalyst batches. (c) HPLC results for the concentration of oxygenate products for CO_2_ fixation with 10 wt % Co/M–SBA-15 catalysts (M = Mg, Al, Ti) collected after 72 h time-on-stream. Adapted with permission from ref (51). Copyright 2023 American Chemical Society.

The performance of these catalysts was investigated in a continuous flow reactor operated at 180 °C and 20 bar (H_2_/CO_2_ = 2:1). As seen in Figure 6a, the evaluation of the catalytic activity of Co/SBA-15 at different Co loadings showed an increase in CO_2_ conversion from 2.0% to 7.2% and 11% with increasing loading from 5 to 10 and 20 wt % Co, respectively. CH_4_, methanol, and CO were detected as the main products along with traces of C_2+_ hydrocarbons (<1%). This set of products is similar to hydrothermal vent chemicals.^53^ All tested catalysts with modified supports were less active in comparison with a nonmodified SBA-15 support and yielded CH_4_ as the main product with selectivity in the range of 56–81%, as shown in Figure 6b. The other products were again methanol, CO, and small amounts of C_2+_ hydrocarbons with different selectivities. The selectivity to methanol was increased when SBA-15 was modified with Zr and Ti. The selectivity to methanol is of particular interest since methanol can also be used as a methyl donor in microbial metabolic pathways.^54^

The collected liquid products contain biomolecules such as formate, acetate, and ethanol (Figure 6c). These oxygenates were also detected with Co–Fe catalysts, showing the ability of Co to convert CO_2_ into metabolic intermediates.^55^ The detection of acetate is of importance since it is an intermediate of the acetyl-CoA pathway. Catalysts with modified SBA-15, particularly those with Ti, exhibited enhanced acetate production, reaching up to 1.2 mM, compared to the unmodified SBA-15 (Co/SBA-15). As methanol plays a crucial role in the metabolic pathway as a methyl donor, CO_2_ hydrogenation was further investigated through a machine learning (ML) approach to enhance methanol selectivity.^56^ The Sure-Independence Screening and Sparsifying Operator model suggested that the reducibility of Co and the adsorption strength of intermediates are the primary features. Based on the results, the model predicted vanadium and zinc as cations for higher methanol selectivity, which was confirmed by experimental results. This indicates that ML could provide insights for predicting active and selective chemical elements for prebiotic CO_2_ fixation at hydrothermal vents. Based on the chemisorption together with product analyses, the reaction proceeds to form a methoxy (*CH_3_O) intermediate that can be desorbed as CH_3_OH or CH_4_ depending on the catalysts.^57^

## 3 Summary and Outlook

A variety of catalysts have been studied for CO_2_ fixation to prebiotic intermediates, encompassing free-standing nanoparticles and supported catalysts. Free-standing NiFe alloys of various compositions demonstrated the conversion of CO_2_ to formate, acetate, and pyruvate—intermediates of the acetyl-CoA pathway. The most active composition, Ni_3_Fe, could further convert pyruvate to citramalate under physiologically viable conditions. NH_3_ treatment of the Ni_3_Fe catalyst resulted in a well-defined Ni_3_FeN/Ni_3_Fe heterostructure, capable of converting CO_2_ and H_2_O into prebiotic molecules such as formamide and acetamide.

CoFe alloys supported on SiO_2_ exhibited similar product profiles to the NiFe system. However, the gas phase analysis revealed the presence of long-chain hydrocarbons up to C_6_. The addition of potassium to the SiO_2_-supported CoFe catalyst influenced particle size and reaction pH, enhancing formate concentration. Further studies under diminished H_2_O concentration involved Co-loaded SBA-15 SiO_2_ modified with cations. Cations successfully modified the surface of SBA-15, leading to partially oxidized Co species after reduction. Although these hardly reducible Co species resulted in less CO_2_ conversion, selectivity to metabolically important methanol was increased noticeably. Those results suggest geochemical CO_2_ fixation at hydrothermal vents could pave the way for the biochemical metabolism performed by enzymes.

Future research will focus on exploring prebiotically plausible heterogeneous catalysts for synthesizing metabolites and biomolecules. This will include investigating other transition metals and their alloys supported on metal oxides found at hydrothermal vents. The goal is to integrate inorganic catalysts and further understand the origins of life, with a particular emphasis on the synthesis of large organic molecules, long-chain alcohols, and carboxylic acids, and the incorporation of elements such as nitrogen, sulfur, and phosphorus into organic molecules. Along with exploring new catalysis, understanding reaction mechanisms is essential in the synthesis of large biomolecules. Some intermediates and products can be synthesized through pathways different from biological ones, providing insights into the prebiotic synthesis of complex biomolecules. To achieve this goal, collaboration in a multidisciplinary approach is highly desirable to look into different aspects of CO_2_ fixation to prebiotic intermediates. This encompasses not only the fields of biology and chemistry but also cutting-edge approaches such as machine learning for catalyst design, allowing for the anticipation of activity and selectivity toward target molecules.
